# Seeds, browse, and tooth wear: a sheep perspective

**DOI:** 10.1002/ece3.2241

**Published:** 2016-07-14

**Authors:** Anusha Ramdarshan, Cécile Blondel, Noël Brunetière, Arthur Francisco, Denis Gautier, Jérôme Surault, Gildas Merceron

**Affiliations:** ^1^ iPHEP UMR 7262 (CNRS & Université de Poitiers)86073Poitiers Cedex 9France; ^2^ Institut P'prime UPR 3346 (CNRS ENSMA & Université de Poitiers)86962Futuroscope Chasseneuil CedexFrance; ^3^ Ferme Expérimentale du MourierInstitut de l'Elevage87800St Priest LigoureFrance; ^4^ Centre Interrégional d'Information et de Recherche en Production OvineFerme Expérimentale du Mourier87800Saint Priest LigoureFrance

**Keywords:** Controlled‐food trials, dental microwear texture analysis, diet, enamel, ruminants

## Abstract

While grazing as a selective factor towards hypsodont dentition on mammals has gained a lot of attention, the importance of fruits and seeds as fallback resources for many browsing ungulates has caught much less attention. Controlled‐food experiments, by reducing the dietary range, allow for a direct quantification of the effect of each type of items separately on enamel abrasion. We present the results of a dental microwear texture analysis on 40 ewes clustered into four different controlled diets: clover alone, and then three diets composed of clover together with either barley, corn, or chestnuts. Among the seed‐eating groups, only the barley one shows higher complexity than the seed‐free group. Canonical discriminant analysis is successful at correctly classifying the majority of clover‐ and seed‐fed ewes. Although this study focuses on diets which all fall within a single dietary category (browse), the groups show variations in dental microwear textures in relation with the presence and the type of seeds. More than a matter of seed size and hardness, a high amount of kernels ingested per day is found to be correlated with high complexity on enamel molar facets. This highlights the high variability of the physical properties of the foods falling under the browsing umbrella.

## Introduction

Feeding habits among ruminants, and more generally among ungulates, have traditionally been divided into three broad categories: grazers, browsers, and mixed feeders (Hofmann 1989). Browsers eat both the woody and the nonwoody parts of dicotyledonous plants, grazers eat predominantly or exclusively monocotyledonous herbs (grasses, sedges rushes), whereas mixed feeders are involved in both grazing and browsing. Browsing, together with the incorporation of fruits and seeds, is thought to be the primitive diet of ungulates (Bodmer and Ward 2006; Janis 2008; but see DeMiguel et al. 2008), before the expansion of grasslands in the late Cenozoic. This hypothesis is primarily based on their low crowned cheek teeth which are thought to be less resistant to the wear inflicted by abrasive diets. It was therefore thought to indicate a softer and less abrasive diet such as browsing (Janis et al. 2002).

While grazing as a selective factor towards hypsodont dentition on mammals has gained a lot attention among paleontologists and evolutionary biologists (Damuth and Janis 2011 and citations therein), seed‐ and fruit‐eating among the browsing ecodietary space has surprisingly been paid less attention. This could be due to the high heterogeneity of items which compose browse. For instance, giraffes (*Giraffa camelopardalis*) and moose (*Alces alces*) are both leaf‐dominated browsers and are grouped together with roe deer (*Capreolus capreolus*) browsing on both foliages and fruit/seeds and even sometimes with highly fruit‐dependent duikers (*Cephalophus* spp.), which are classified into their own ecodietary space as frugivores by Gagnon and Chew (2000). Fruits and seeds are an important piece in the dietary ecology of several ungulates inasmuch as they constitute an essential fallback resource (less preferred foods) for many taxa in times when preferred foods are scarce (Marshall and Wrangham 2007). For example, in modern roe deer, blackberries play a major role in summer as a significant dietary supplement for roe deer; both for nursing females as well as for roebuck fully invested in the rutting period. Later in late autumn and winter, acorns are key fallback foods as the vegetal resources are low during that period and because roebuck recover from a second rutting period (Duncan et al. 1998; Cransac et al. 2001). The importance of fruits and seeds can be seen in their role in the ecological niche partitioning among frugivorous species (defined here as species foraging mostly on both fruits and seeds, the latter being included in the former). For instance, size of fruits and seeds fallen from the upper canopy together with nycthemeral rhythms are the two main factors controlling the niche overlapping for sympatric duikers in Central Africa (Heymans and Lejoly 1981; Feer 1988; Wilson 2005). Therefore, seeds and fruits might be critical for population demography of many species of ungulates (Massei et al. 1996). To detect and assess the weight of seed‐ and fruit‐eating in the evolutionary history of mammals is therefore important.

Tooth wear reflects individual senescence, availabilities of food resources, and niche partitioning among species of mammals (Janis 1990; Nussey et al. 2007; Kaiser et al. 2013; Calandra and Merceron 2016). From the scale of a whole tooth to the micrometric scars on dental facets, differences in dietary preferences are mirrored through tooth wear analysis. When focusing on the dietary categories of browsing species, dental facet patterns and dental mesowear discriminate ruminants highly engaged in frugivory such as duikers from other browsing species (Janis 1990; Fortelius and Solounias 2000; Kaiser et al. 2013). When looking at earlier studies in 2D dental microwear analysis, densities of scratches and of large pits and scratches have been shown to discriminate leaf‐browsing species such as the giraffe, mixed (fruit and foliage) browsers such as the roe deer and fruit‐browsing species such as duikers (Solounias and Semprebon 2002; Merceron et al. 2007, 2012). However, these semi‐quantitative methods have an Achilles’ heel: the lack of repeatability (especially between observers; Grine et al. 2002; Galbany et al. 2005; Mihlbachler and Beatty 2012; Mihlbachler et al. 2012; personal observations; for detailed reviews of intra‐ and interobserver errors, see DeSantis et al. 2013). An alternative methodology has provided automated, repeatable, and quantitative characterizations of 3D surfaces free of observer measurement errors (Scott et al. 2006; DeSantis et al. 2013).

Dental Microwear Textural Analysis (DMTA hereafter) has proved to be particularly useful in assessing diets of fossil as well as modern taxa (Calandra and Merceron 2016; Haupt et al., 2013; Merceron et al. 2007, 2014; Scott et al. 2006; Souron et al. 2015; Ungar et al. 2007). Among modern ungulates, the only mammals that have been extensively studied are the African antelopes (Ungar et al. 2007; Scott 2012). A preliminary ecodietary space based on dental microwear texture can be pictured. In her study of African bovids, Scott (2012) shows that browsers have high complexity, textural fill volume, and heterogeneity and a low anisotropy when compared to grazing antelopes. Also, Scott (2012) has found that frugivores (all cephalophini) differ from other browsing species (including leaf browsers and leaf/fruit browsers) in all variables except anisotropy. Merceron et al. (2010) has reached the same conclusion when exploring feeding behavior throughout the year and depending on gender among a modern population of roe deer. Indeed, roebuck shot in winter while foraging a lot on fallen acorns have higher complexity of dental microwear textures than females browsing mostly on brambles semi‐persistent and mature leaves. Such differences are also supported when comparing the dental microwear textures between the leaf‐browsing giraffe and the frugivorous yellow‐back duiker (Merceron et al. 2014). Although the advent of DMTA has allowed for more precise and repeatable dietary reconstructions (DeSantis et al. 2013), the root causes for the formation of microwear patterns remain to be characterized in a controlled setting (Hua et al. 2015; Xia et al. 2015). Controlled‐food experiments, by reducing the dietary breadth, allow for a direct quantification of the effect of each type of items separately (i.e., seed vs. browse vs. graze). The few previous studies have not focused on frugivory and granivory but on diet abrasiveness with a 32‐rabbit model (Schulz et al. 2013) and the phytolith/dust debate with a 4‐ewe model (Hoffman et al. 2015).

In this study, we present the results of a DMTA on 40 ewes (*Ovis aries*) under a controlled‐food experimentation. This study aims to assess the effects of seed amount, size, and hardness in complement with a leaf‐browse diet on dental microwear texture. Following the preliminary results from the previous DMTA studies (Ungar et al. 2007; Merceron et al. 2010, 2014; Scott 2012), we would expect the following: (1) The complexity (*Asfc*), the textural filling volume (*Tfv*), as well as the heterogeneity of complexity (*HAsfc*) to be higher and the scale of maximum complexity (*Smc*) to be lower for ewes fed with seeds as complement with clover. (2) The complexity (*Asfc*), the textural filling volume (*Tfv*), the heterogeneity of complexity (*HAsfc*) as well as the scale of maximum complexity (*Smc*) to be correlated with seed density, size and hardness. (3) The anisotropy (*epLsar*) to be similar between the four groups. (4) Canonical discriminant analysis based on dental microwear textural parameters to correctly classify *a posteriori* ewes in their respective diet.

## Material and Methods

### Experimental conditions

The controlled‐food trials were carried out at the Mourier farm (Limousin region, France; agreement number B‐87‐176‐01), under the supervision of the *Centre Interrégional d'Information et de Recherche en Production Ovine* (CIIRPO) and the *Institut de l'Elevage* (Idele). G.M. and D.G., who have official approval to carry out such procedures, designed these trials. They were performed on domestic sheep (*Ovis aries*), using only ewes from the *Vendéen* breed. All experiments were conducted on cull ewes, meaning sheep no longer suitable for breeding and sold for meat. None of the ewes were put down solely for the purpose of the experimentation. None of the experiments required the sheep to be handled. Sheep had full access to foods with which they were familiar. The sheep were kept inside a covered sheep hold, and fed during a minimum period of 70 days from 15 July 2014 to 2 October 2014 for the ewes fed on clover alone and from 20 November 2014 to 6 February 2015 for the three groups fed with clover and a complement of seeds. The sheep were not kept on hay, which they would have eaten, but rather on dust‐free wood shavings. Feeding troughs were covered with a plastic film and cleaned out daily to avoid contamination. Forty sheep were included in this study, divided into four groups of ten (Appendix S1). Each group corresponds to a different diet: clover silage with chestnuts, barley, or corn (i.e., grains of different size and hardness; Table 1) and one group fed exclusively on clover silage. Each of the first three 10‐ewe groups was fed 4.25 kg (i.e., 25% of the diet as dry matter weight) of grains/seeds daily, together with access to 12.75 kg (i.e., 75%) of fodder. These amounts were defined by how much the ewes had consumed in 24 h during a 5‐day period of adaptation to the diet. Ewes have consumed the daily whole portion. None of the ewes lost weight during the experiments.

**Table 1:** Summary statistics (mean m and standard deviation SD) for density of seeds, hardness index, and length of major and minor axis for each type of seed: barley, corn, and chestnut. Densities in seeds per kg, measurements in mm (major and minor axes), and hardness indexes in Newton N (Fox et al. 2007; Singh and McCain 1963; Yildiz et al. 2009).Twenty seeds per species were considered for size measurements

Seeds	Density	Hardness		Major axis		Minor axis	
		m	SD	m	SD	m	SD
Barley	~10,000	61.69	5.82	10.86	1.28	3.22	0.22
Corn	~2500	122.36	10.47	11.96	1.24	5.61	0.94
Chestnuts	~100	77.05	6.92	33.51	4.11	27.06	2.85

The fodder was harvested from a 2.5‐ha field highly sown with red clover (*Trifolium pratense*) in September 2013. The red clover silage is composed of 88% dicots (72% of red clover *Trifolium pratense*) and 12% herbaceous monocots, mostly *Lolium hybridum*. In early July 2014, after 81 mm of precipitations spread over 23 June 2014 to 5 July 2014, the field was cut 10 cm above the ground to avoid including grit in the harvest and was bale‐wrapped 24 h after the cutting in order to guarantee similar natural physical properties (percentage of dry matter about 50%) to the uncut plant throughout the experimentation. Also, due to the precipitations that occurred, the harvest was expected to be free of air‐born dust. This has been double‐checked by counting the endogenous mineral bodies versus exogenous elements after mineralization by incineration and acid attacks on the red clover. More than 90% of the elements issued from the residues are not dust but endogenous organic minerals (silica phytoliths from the few grasses incorporated within the clover silage).

As planned by the Mourier farm, cull ewes were sold for meat after the 70 days experimentation. Due to sanitary and veterinary regulations in the slaughterhouse, stomach content could not be sampled.

### Preparation and casting

**Figure 1:**
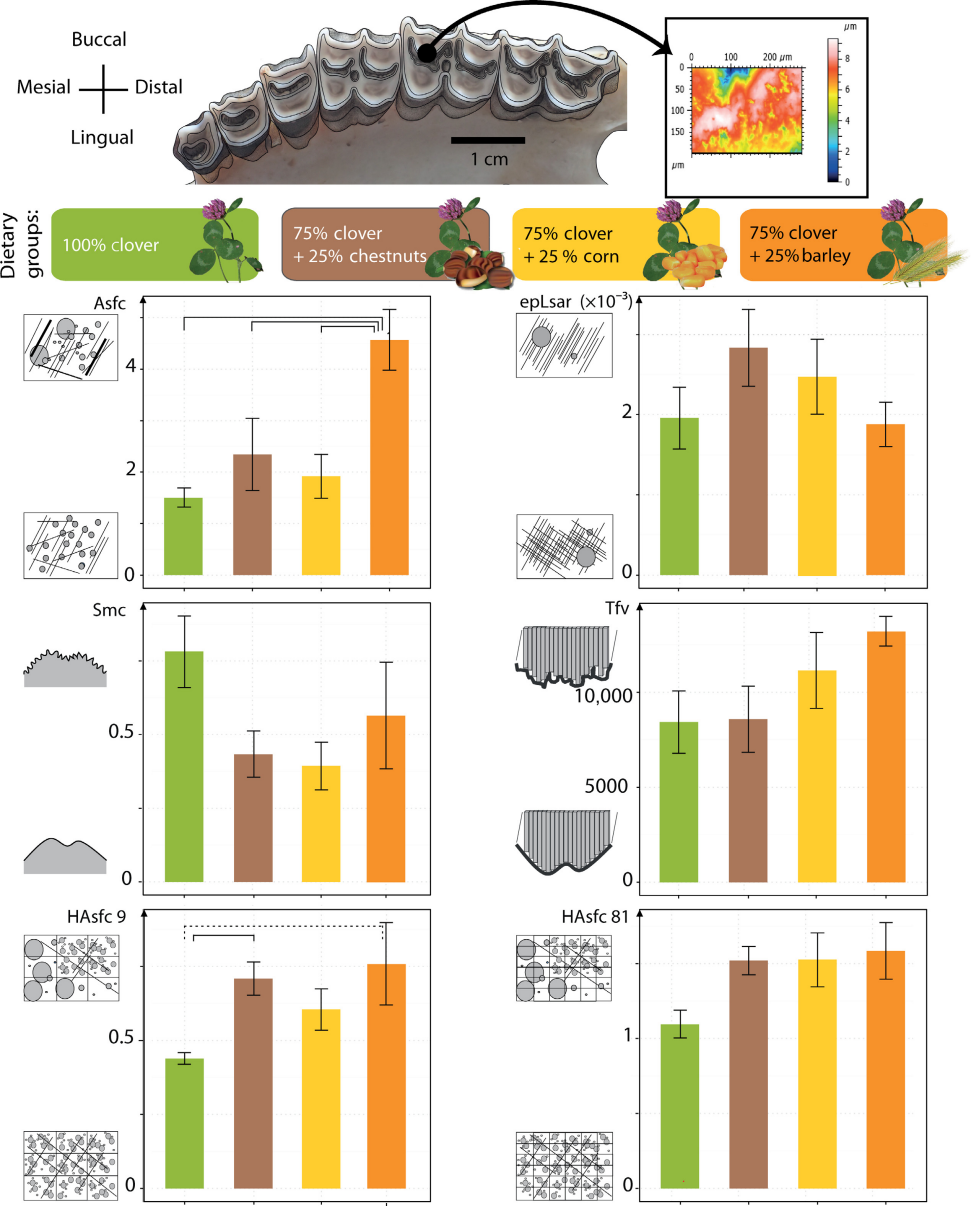
From teeth to dental microwear textural parameters. Upper left cheekteeth of a modern sheep (*Ovis aries*). The scanning of the lingual enamel band of the paracone on the second molar allows running dental microwear texture analysis (A). Barplots of the mean and standard error of the mean of the six parameters for each dietary category. *Asfc*, complexity; *EpLsar*, anisotropy; *Smc*, scale of maximum complexity; *Tfv*, Textural fill volume; *HAsfc*_9_ and *HAsfc*_81_, heterogeneity for 3 × 3 and 9 × 9 cells, respectively. Significant differences between groups are noted with horizontal braces and with dotted‐line braces if the both LSD and HSD tests detect significant differences.

The skulls were prepared following standard procedures in osteological preparation (Jakway et al. 1970). Each tooth was carefully cleaned. The facet is located on the disto‐ or mesio‐lingual enamel band of the paracone of the upper left second molar (Fig. 1). Molds are then made using a polyvinylsiloxane elastomer (Regular Body President, ref 6015 ‐ ISO 4823, medium consistency, polyvinylsiloxane addition type; Coltene Whaledent). This product is known to be the most efficient one to replicate a given surface at fine scales (Galbany et al. 2006; Goodall et al. 2015).

The molds are then placed under a Leica DCM8 confocal profilometer using white light confocal technology with a Leica 100× objective (NA = 0.90; working distance = 0.9 mm). The center of the dental shearing facet of interest was sampled. Surface elevations for each specimen were collected at a lateral (*x*, *y*) interval of 0.129 *μ*m with a vertical numerical step of 1 nm. For each specimen, a total surface of 333 × 251 *μ*m (2584 × 1945 points; Fig. 1) is scanned. These raw data are then mirrored to obtain the true relief of the enamel surface and compiled in LeicaMap. From the whole surface, four adjacent sub‐surfaces (140 × 100 *μ*m; Fig. 1; Appendix S2) were generated in order to be consistent with the procedures shown in Scott et al. (2006) and widely used as standard by many colleagues (e.g., Scott 2012; Scott et al. 2012; Stynder et al. 2012; Donohue et al. 2013). Data were analyzed with a scale‐sensitive fractal analysis using Toothfrax and Sfrax software (Surfract, www.surfract.com) following Scott et al. (2006).

### Data analysis

Five microwear variables are used in this study (Table 2; Appendix S1). Complexity (*Asfc* or area‐scale fractal complexity) is a measure of the roughness at a given scale. The scale of maximum complexity (*Smc*) quantifies the range over which *Asfc* is calculated. Anisotropy (*epLsar* or exact proportion of length‐scale anisotropy of relief) measures the orientation concentration of surface roughness. Textural fill volume (*Tfv*) is the result of an algorithm that fills a surface with square cuboids of different volumes. *Tfv* does not depend on the surface shape but on its finer texture. Another variable has been proved to be useful in differentiating diets. Heterogeneity of complexity (*HAsfc* or heterogeneity of area‐scale fractal complexity), quantifies the variation of complexity observed within a given scan. *HAsfc* is calculated in each 140 × 100 *μ*m subsurface through 9 and then 81 cells (*HAsfc*_9_ and *HAsfc*_81_, respectively). All variables have been described in further detail in Scott et al. (2006). Statistical tests were then used in order to highlight potential differences in dental microwear textural parameters between the dietary groups. As textural parameters violated conditions for parametric tests, they were rank‐transformed before each analysis (Sokal and Rohlf 1969; Conover and Iman 1981). Single‐classification ANOVAs for each parameter were used to determine the sources of significant variation. Any potential difference was then highlighted using the combination of the conservative HSD test (Tukey's Honest Significant Differences) together with the less conservative LSD test (Fisher Least Significant Differences; Fig. 1; Table 3). A first look at the variations in complexity with age displays no direct relation (Appendix S3).

**Table 2:** Mean (m) and standard deviation (SD) for microwear textures variables according to dietary group. *Asfc*: area scale fractal complexity (no unit), *epLsar*: length‐scale anisotropy of relief (×10^−3^; no unit), *Smc*: scale of maximum complexity (*μ*m^2^), *HAsfc*_9_ and *HAsfc*_81_: heterogeneity of area‐scale fractal complexity on 3 × 3 or 9 × 9 cells (no unit), *Tfv*: textural fill volume (*μ*m^3^). All variables have been described in further detail in previous studies (Scott et al. 2006)

Dietary groups	*Asfc*		*epLsar*		*Smc*		*HAsfc*_9_		*HAsfc*_81_		*Tfv*	
	m	SD	m	SD	m	SD	m	SD	m	SD	m	SD
Clover + Chestnuts	2.34	2.21	2.83	1.52	0.43	0.25	0.71	0.18	1.52	0.30	8588.2	5506.5
Clover + Corn	1.92	1.34	2.48	1.48	0.39	0.26	0.60	0.22	1.52	0.57	11139.4	6326.8
Clover + Barley	4.57	1.88	1.88	0.87	0.57	0.57	0.76	0.44	1.58	0.60	13215.7	2479.6
Clover	1.50	0.58	1.96	1.22	0.78	0.38	0.44	0.06	1.10	0.29	8439.3	5202.2

**Table 3:** Results for the statistical tests. (a) Single‐classification ANOVAs with ranked data on textural parameters, (b) Tukey's HSD (below the diagonal) and Fisher's LSD (above the diagonal) pairwise comparison test on ranked data for complexity *Asfc* and heterogenity of complexity *HAsfc*_9_

		df	SS	MS	*F*	*P*
(a)						
*Asfc*	Effect	3	2099.60	699.87	7.799	**3.88×10** ^**−4**^
	Error	36	3230.40	89.73		
*epLsar*	Effect	3	423.80	141.27	1.037	0.388
	Error	36	4906.20	136.28		
*Smc*	Effect	3	754.65	251.55	1.991	0.133
	Error	36	4549.35	126.37		
*HAsfc*_9_	Effect	3	1235.80	411.93	3.622	**0.022**
	Error	36	4094.20	113.73		
*HAsfc*_81_	Effect	3	994.60	331.53	2.753	0.056
	Error	36	4335.40	120.43		
*Tfv*	Effect	3	605.80	201.93	1.539	0.221
	Error	36	4724.20	131.23		

	Clover + chestnuts	Clover + Corn	Clover + Barley	Clover
(b)				
*Asfc*				
Clover + chestnuts		0.742	**1.5×10** ^**−3**^	0.351
Clover + Corn	0.987		**5.8×10** ^**−4**^	0.543
Clover + Barley	**7.7×10** ^**−3**^	**3.2×10** ^**−3**^		**9.6×10** ^**−5**^
Clover	0.781	0.927	**6.6×10** ^**−4**^	
*HAsfc*_9_				
Clover + chestnuts		0.134	0.341	**0.003**
Clover + Corn	0.430		0.574	0.102
Clover + Barley	0.770	0.941		**0.031**
Clover	**0.014**	0.350	0.131	

df, degrees of freedom; SS, sum of square; MS, mean square; *F,* Fisher value; *P, P*‐value.

**Figure 2:**
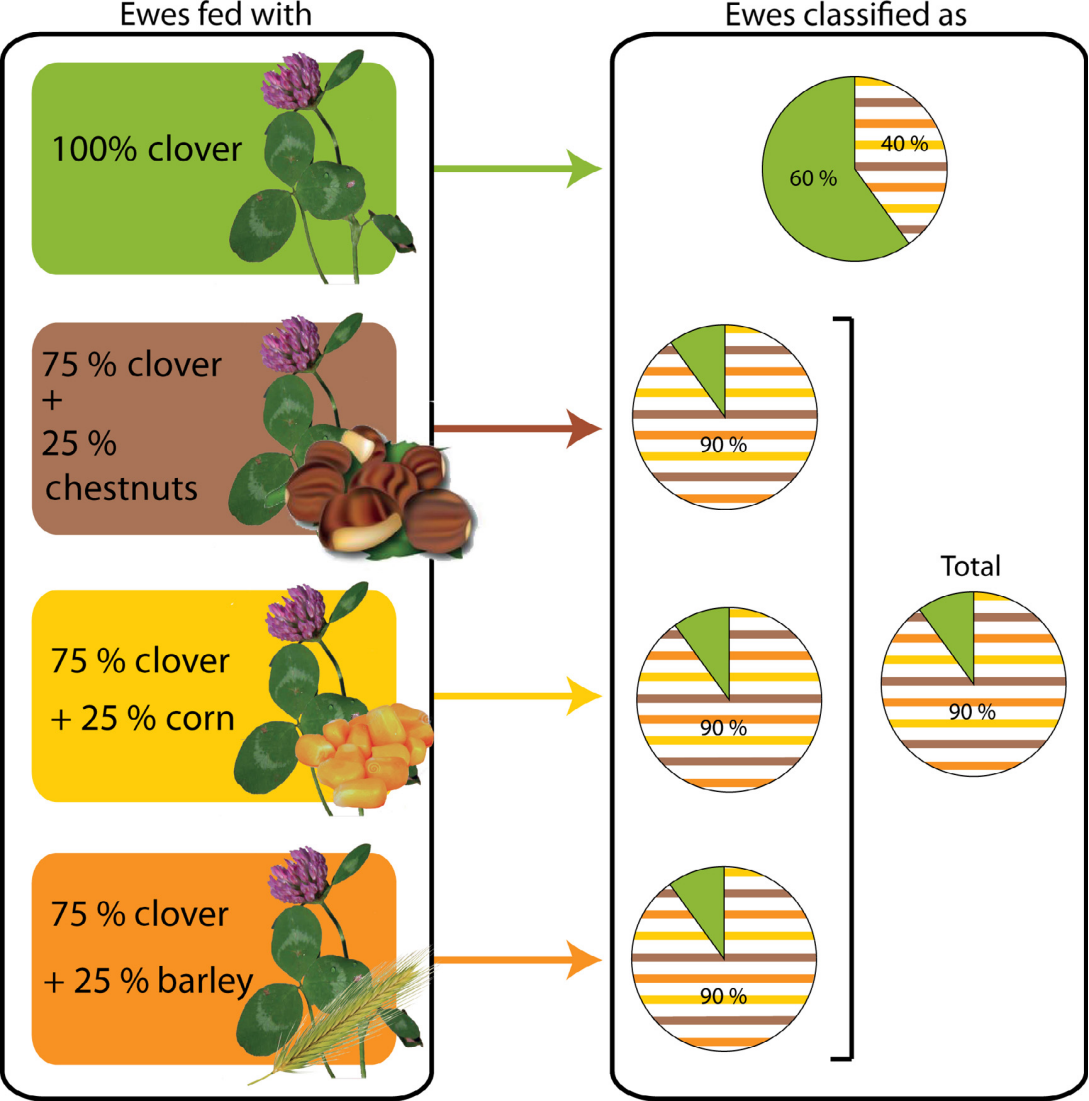
Classify into the browsing ecodietary spaces. The canonical discriminant analysis set up with 40 ewes clustered in two a priori groups (seed‐free and clover‐fed ewes, and seed‐ and clover‐fed ewes) and five dental microwear textural parameters allows a sharp *a posteriori* (Jackknifed) classification with 90% of the seed‐fed ewes well classified (online version in color).

In some cases, a species might be assigned to a dietary category based on a given parameter but plots with another one when a second parameter is considered. Combining all of these parameters into a set of discriminant functions may offer some help in dietary classification. Two canonical discriminant analyses including a Jackknife resampling procedure for classification were then run with five textural parameters. To avoid the overweighting of the heterogeneity of complexity, we made the choice here to discard *HAsfc*_81_ and to focus on *HAsfc*_9_. A first analysis clustered the ewes in two groups (defined as seed and seed‐free diets) and a second includes the four groups of ewes (Fig. 2; Table 4; Appendix S4). The open‐source software R (R Core Team, 2015) was used with the following packages: MASS (Venables and Ripley 2013), plyr (Wickham et al. 2011), agricolae (De Mendiburu 2014), and psych (Revelle 2014).

**Table 4:** Canonical discriminant analysis data. Raw (raw coeff.) and standardized (st. coeff) coefficients of the discriminant function (DF), and *a posteriori* classification with and without Jackknifed resampling procedure comparing clover‐fed and seed‐fed groups (a; chestnut, corn and barley grouped together) and comparing the four dietary categories (b)

	DF1‐raw coeff.	DF1‐st. coeff.
(a)		
*Asfc*	−0.225	−0.425
*epLsar*	−376.120	−0.493
*Smc*	1.659	0.637
*HAsfc*_9_	−2.176	−0.574
*Tfv*	7.06×10^−6^	0.036

Groups	*N*	Predicted dietary category (with/without Jackknifed resampling procedures)		Correctly classified (%)
		Clover	Seeds	
Clover	10	6/6	4/4	60/60
Clover + Seeds	30	4/3	26/27	86.7/90

	LD1‐raw coeff.	LD1‐st. coeff.	LD2‐raw coeff.	LD2‐st. coeff.	LD3‐raw coeff.	LD3‐st. coeff.
(b)						
*Asfc*	−0.508	−0.826	−0.032	−0.052	0.289	0.470
*epLsar*	151.959	0.197	−568.949	−0.739	278.079	0.361
*Smc*	−0.673	−0.262	2.084	0.809	0.981	0.382
*HAsfc*_9_	−0.942	−0.249	−2.016	−0.533	0.239	0.063
*Tfv*	−5.14×10^−5^	−0.261	−7.23×10^−5^	0.368	1.85×10^−4^	−0.939
% variance	0.553		0.3782		0.0688	

Groups	*N*	Predicted dietary category (with/without Jackknifed resampling procedures)				Correctly classified (%)
		Clover	Chestnuts	Corn	Barley	
Clover	10	7/8	1/1	2/1	0/0	70/80
Clover + Chestnuts	10	1/1	5/6	3/2	1/1	50/60
Clover + Corn	10	2/2	3/2	3/4	2/2	30/40
Clover + Barley	10	1/1	1/0	2/2	6/7	60/70

## Results

Results indicate significant differences between groups for *Asfc* and *HAsfc*_9_. The ANOVAs do not detect any significant differences in *Smc*,* epLsar*,* HAsfc*_81_, and *Tfv* (Fig. 1; Table 3). Only the ewes fed on barley significantly differ from clover seed‐free fed groups. Moreover, the sheep that were fed with chestnuts and in lesser extent those fed on barley show a significantly higher heterogeneity of complexity (*HAsfc*_9_) than the ewes that ate clover alone (Tables 2 and 3, Fig. 1).

The canonical discriminant analyses assessing the rate of a posteriori misclassification of ewes in their respective dietary ecospace provide contrasting results depending on the analysis (Fig. 2; Table 4, Appendix S4). With 82.5% of success rate in classification (80% with Jackknifed classification; Table 4, Appendix S4), the 2‐group analysis makes it clear that dental microwear texture highlights variations in food preferences within the browsing ecodietary space. Ninety percent of the ewes (86.7%; with Jackknifed classification; Table 4, Fig. 2, Appendix S4) fed on clover with complement of seeds are correctly classified. Only 60% of the seed‐free and clover‐fed ewes are correctly classified. The rates of classification drop when the 4‐group analysis is performed (Fig. 2; Table 4, Appendix S4). It is worth to mention that although the two parameters *epLsar* and *Smc* do not show significant differences between groups, their contributions to the canonical discriminant functions are as high as those which significantly differ between groups (*Asfc* and *HAsfc*_9_; Table 4).

## Discussion

This study highlights differences in dental microwear textures between ewes having fed on foods emphasizing differences into the browsing category. The quantity of seeds given to these ewes is much lower than the one eaten by frugivorous species such as the African duikers which may include more than 80% of fruits (with seeds) in its diet (Gagnon and Chew 2000 and citations therein). Twenty‐five percents of seeds expressed as dry matter weight of a daily dietary bolus makes the three groups of seed‐eating ewes fall within the browsing category senso Gagnon and Chew (2000). However, variations within this dietary ecospace are here found through DMTA.

### DMTA to track dietary variations among browsers

In the wild, browse diets are naturally more variable in size and shape with larger, harder, and more brittle items requiring more crushing expected to lead to pits of a variety of shapes and sizes (Ungar et al. 2007; Scott et al. 2012). Seeds need to be crushed in order to gain access to the softer inner endosperm and cotyledon, the strength of the crushing required to fracture the outer seed coat depending on the seed hardness. Hardness is characterized by the hardness index, meaning the strength required to generate a plastic deformation on a given item (Lucas 2004). The compression of harder material will generate higher stress in the tooth (Johnson 1985) which in turn will help in crack propagation and wear of the tooth (Keer et al. 1982). Food hardness has been linked to texture complexity in previous studies (Scott et al. 2012; Souron et al. 2015), as harder items require more force during mastication to be processed. In this hypothesis, the group showing the highest complexity should also correspond to the hardest dietary item. Barley is softer than corn (Singh and McCain 1963; Fox et al. 2007; Yıldız et al. 2009), and the ewes that were fed barley show the highest complexity values. In this case, the seed hardness is not the determining factor to generate complex surface on enamel. However, we did not here consider the particle size after mastication but at the seed size itself.

Another factor to consider is the food particle size because, as reminded by Keer et al. (1982), the smaller the particles, the higher the wear. Furthermore, Lucas et al. (2008) have shown that large hard particles have the potential to fracture enamel, whereas small hard particles can only indent it. As such, independently of texture complexity, wear (defined as the loss of tooth tissue) would be greater with large particles. It has been proposed that certain microwear features such as “ovoid pits” (indentation of enamel) might be linked to the consumption fruits including small seeds (Solounias and Semprebon 2002). The groups showing the highest complexity were given barley kernels (Table 1; Fig. 1), which are the smallest kernels of this study. A smaller seed size also implies more grains are taken per mouthful, which in turn would imply more pitting, as there are more small seeds to be crushed. Indeed, the density of barley approximates 10,000 kernels/kg, compared to 2500 for corn and 100 for chestnuts (Table 1). Therefore, smaller seed leads to more loading cycles on the tooth surfaces and enhance fatigue wear (Keer et al. 1982). Also, the digestibility of barley is known to be low (McAllister et al. 1994), as the hull and the pericarp of whole barley grains are very resistant to bacterial attachment and digestion in the rumen. Barley does therefore require additional processing by the teeth, which could explain the higher complexity values in dental microwear textures seen among this dietary group. Available data on the digestibility of chestnuts focus principally on the tannin content rather than on the mechanical properties of the chestnut. Similarly, available data in the literature focus on the chemical properties of corn. Starch utilization can be enhanced by additional processing in ruminants, but has little impact in sheep (Theurer 1986).

Heterogeneity in complexity across a surface has been shown in previous studies to be of value for distinguishing microwear patterns (Scott 2012; Souron et al. 2015). As expected, a monotypic diet such as seed‐free diet generates homogeneous textures across a dental facet when compared with polytypic diets. Here, Chestnut‐fed and barley‐fed sheep have values of heterogeneity of complexity significantly higher than those fed on clover only. Corn do not generate significantly higher values of heterogeneity on enamel than clover and seed‐free diet. Barley kernels, corn kernels, and chestnuts differ in size, inner content, and shape, so we also expect differences in heterogeneities of complexity depending on seeds.

We also expected this study to highlight differences in the scale of maximum of complexity (the scale at which the complexity is calculated) between the groups that fed on clover and those that fed also on grains. No such difference was highlighted in this study. *Smc* has been linked to the scale of the wear‐causing particles (Scott 2012). Our results cannot distinguish between seed‐free and seed‐eating ewes based on *Smc* alone.

Although there is no significant difference among the four groups of this study, it is worth to mention that there is a negative correlative trend between *Tfv* and seed size. Smaller is the seed size, higher is *Tfv*. Barley produced more texture relief at lower scale (higher *Tfv*) than chestnuts or corn, or even more than the mere consumption of seed‐free clover diet.

Browse and seeds, such as the food types featured in this study, are reduced by crushing motions rather than sheared through like grass. These food types are therefore not conducive to the formation of scratches and result in microwear textures with low anisotropy values (below 3.0 × 10^−3^) as illustrated by the results of this study. However, earlier studies have shown that species browsing on both foliages and fruit/seeds display more scratches than leaf‐dominated eaters (Solounias and Semprebon 2002; Merceron et al. 2012). Our results reveal a more complex pattern. Sheep fed on clover and barley have values of anisotropy as low as sheep fed on clover alone simulating leaf‐browsing habits.

### Canonical discriminant analysis as a model to frame the browsing ecodietary space

One of the main issues in paleoecology is to assign extinct taxa into a given category, whereas there is no discrete category but a continuum. A way to better frame the feeding ecology of the extinct mammals is to use canonical discriminant analysis in combination with Jackknifed classification.

When looking at the 2‐group canonical discriminant analysis on sheep under controlled‐food testing, we may conclude that browsers involved in leaf‐ and fruit‐eating (seeds being included into fruits) discriminate from leaf browsers (here simulated by ewes that were only fed clover; Fig. 2). Seeds affect dental microwear texture even though the amount is thrice lower than the amount of foliages into the dietary bolus. The rate of misclassification for seed‐free and clover‐fed ewes is higher compared with that of ewes fed on grains (Table 4; Fig. 2). We may then hypothesize that when at least 50% of the sample falls into the leaf‐browsing ecodietary space (defined by low values for complexity, anisotropy, and heterogeneity of complexity), a given species with unknown a priori diet might be assigned to the leaf‐browsing ecodietary space.

When looking at the 4‐group discriminant analysis (Table 4; Fig. 2), to picture the link between dental microwear textures and properties of seeds, which could be a major factor to avoid ecological overlapping among browsers, seems at best uneasy since dental microwear textural ranges overlap. This result may seem somewhat counterintuitive as barley, chestnuts, and corn have very different grain sizes, densities, and hardness (Table 1).

## Conclusions

The controlled‐food experiment framework aims at detangling the root causes of dental microwear texture. By focusing diet on single dietary items with known physical properties, we can test the influence of factors such as seed size and hardness on dental microwear texture. Although this study focuses on diets which all fall within a single dietary category (browse), the groups show significant differences in dental microwear textures in relation to differences in dietary bolus. Our sets of hypothesis formulated above are partly validated.

First of all, not all samples of seed‐ and clover‐fed ewes have significant differences with seed‐free and clover‐fed ewes. Actually, only barley‐ and clover‐fed ewes have indeed higher complexity and heterogeneity of complexity than the ewes fed only on clover. None of the observed variations in the scale of maximum complexity and in the textural filling volume are significant. Among the seed‐fed ewes, there is no clear relation between seed properties (size, hardness) with any of the textural parameters. More than a matter of seed size and hardness, a high amount of kernels ingested per day, such as the amount of barley (about 5000 units per day and per ewe in this study), is found to be correlated with high complexity on enamel facets. As expected, anisotropy of the dental microwear texture does not vary significantly between groups although its contribution to the discriminant functions is as high as complexity. A posteriori Jackknifed classifications through the canonical discriminant analysis discriminate successfully seed‐free to seed‐eating browsers.

## Conflict of Interest

None declared.

## Supporting information


**Appendix S1**. List of ewes (specimen number and known age) clustered into their dietary groups with individuals raw dental microwear textural parameters.

**Appendix S2**. Photosimulations of the enamel surfaces scanned with the Leica DCM8 surface profilometer for each of the 40 ewes.

**Appendix S3**. *Asfc* distribution according to specimen age and according to dietary group: clover (in green); chestnuts (in red); corn (in blue); barley (in black).

**Appendix S4**. Canonical discriminant analysis. Posterior individual probabilities for the CDA comparing clover‐fed and seed‐fed ewes (a) and comparing the four dietary categories (b).
