# Supplementary material for: Seeds, browse, and tooth wear: a sheep perspective
Source: Ecol Evol. 2016 Jul 14;6(16):5559–69. doi: 10.1002/ece3.2241 (PMC4983574; doi:10.1002/ece3.2241)
Supplement: Supplementary file 1 — Appendix S1. List of ewes (specimen number and known age) clustered into their dietary groups with individuals raw dental microwear textural parameters. [file ECE3-6-5559-s001.docx]

Appendix S1. List of ewes (specimen number and known age) clustered into their dietary groups with individuals raw dental microwear textural parameters.

| Group | Specimen | Age (year) | Asfc | epLsar (×10^-3^) | Smc | HAsfc_9_ | HAsfc_81_ | Tfv |
| --- | --- | --- | --- | --- | --- | --- | --- | --- |
| Clover | 08-045 | 4 | 1.11 | 6.17 | 0.60 | 0.37 | 1.39 | 3002.31 |
| Clover | 10-098 | 3 | 1.33 | 1.50 | 1.02 | 0.58 | 1.14 | 6855.89 |
| Clover | 11-723 | 3 | 1.86 | 8.09 | 1.02 | 0.52 | 0.90 | 11782.64 |
| Clover | 20-939 | 2 | 2.25 | 3.63 | 1.52 | 0.41 | 0.93 | 13035.53 |
| Clover | 31-042 | 1 | 1.07 | 2.20 | 0.92 | 0.42 | 0.86 | 8681.15 |
| Clover | 70-519 | 7 | 1.15 | 2.25 | 0.61 | 0.43 | 1.05 | 1946.47 |
| Clover | 80-140 | 6 | 0.93 | 4.38 | 0.92 | 0.40 | 0.84 | 13496.85 |
| Clover | 80-307 | 6 | 2.69 | 1.89 | 0.13 | 0.44 | 1.12 | 17113.43 |
| Clover | 80-729 | 6 | 1.27 | 1.02 | 0.40 | 0.41 | 1.78 | 3570.85 |
| Clover | 90-287 | 5 | 1.37 | 1.29 | 0.68 | 0.40 | 0.93 | 4908.55 |
| Chestnuts | 07-823 | 4 | 1.56 | 1.76 | 0.13 | 0.79 | 1.67 | 13931.90 |
| Chestnuts | 20-251 | 2 | 1.60 | 4.62 | 0.30 | 0.82 | 1.38 | 10631.29 |
| Chestnuts | 21-269 | 2 | 0.69 | 1.75 | 0.53 | 1.13 | 2.17 | 5709.95 |
| Chestnuts | 30-003 | 1 | 8.23 | 9.60 | 0.13 | 0.58 | 1.32 | 13097.38 |
| Chestnuts | 30-870 | 1 | 3.09 | 3.78 | 0.53 | 0.67 | 1.34 | 12117.23 |
| Chestnuts | 31-051 | 1 | 1.08 | 1.34 | 0.83 | 0.57 | 1.65 | 2042.77 |
| Chestnuts | 80-171 | 6 | 0.99 | 2.10 | 0.41 | 0.73 | 1.14 | 13069.36 |
| Chestnuts | 80-212 | 6 | 1.25 | 3.02 | 0.75 | 0.67 | 1.26 | 454.58 |
| Chestnuts | 80-721 | 6 | 2.07 | 3.42 | 0.54 | 0.63 | 1.60 | 1614.68 |
| Chestnuts | 90-126 | 7 | 2.84 | 5.60 | 0.17 | 0.50 | 1.67 | 13212.97 |
| Corn | 00-063 | 4 | 1.04 | 1.89 | 0.17 | 1.05 | 2.08 | 6748.27 |
| Corn | 21-150 | 2 | 1.43 | 8.16 | 0.27 | 0.66 | 2.65 | 829.01 |
| Corn | 21-315 | 2 | 0.57 | 3.06 | 0.83 | 0.35 | 0.89 | 1568.59 |
| Corn | 30-017 | 1 | 1.58 | 1.98 | 0.25 | 0.61 | 1.65 | 18724.55 |
| Corn | 31-033 | 1 | 4.37 | 1.51 | 0.17 | 0.59 | 1.28 | 17870.20 |
| Corn | 31-075 | 1 | 4.31 | 1.34 | 0.13 | 0.80 | 1.59 | 9490.48 |
| Corn | 80-086 | 6 | 1.44 | 3.36 | 0.49 | 0.37 | 1.13 | 12910.52 |
| Corn | 80-236 | 6 | 2.13 | 1.82 | 0.75 | 0.49 | 1.37 | 13229.77 |
| Corn | 80-403 | 6 | 1.09 | 5.97 | 0.60 | 0.38 | 0.77 | 15112.69 |
| Corn | 90-171 | 7 | 1.20 | 3.08 | 0.25 | 0.74 | 1.84 | 14910.17 |
| Barley | 07-347 | 4 | 2.90 | 3.04 | 0.07 | 0.55 | 0.85 | 14660.38 |
| Barley | 21-253 | 2 | 5.16 | 1.08 | 0.13 | 0.53 | 1.82 | 12098.20 |
| Barley | 21-745 | 2 | 4.77 | 2.45 | 1.00 | 0.95 | 1.87 | 11038.54 |
| Barley | 30-068 | 1 | 7.84 | 2.33 | 0.04 | 1.76 | 2.88 | 15349.22 |
| Barley | 31-045 | 1 | 3.29 | 8.58 | 0.75 | 0.38 | 1.00 | 9934.39 |
| Barley | 31-078 | 1 | 7.26 | 1.23 | 0.13 | 0.35 | 1.51 | 11004.08 |
| Barley | 80-012 | 6 | 3.73 | 2.82 | 1.23 | 1.14 | 1.75 | 15116.18 |
| Barley | 80-369 | 6 | 1.92 | 7.05 | 0.11 | 0.40 | 0.95 | 12599.59 |
| Barley | 80-661 | 6 | 3.64 | 1.66 | 0.53 | 0.64 | 1.38 | 17949.54 |
| Barley | 90-237 | 7 | 5.18 | 2.63 | 1.65 | 0.88 | 1.82 | 12407.60 |
