# Supplementary material for: Seeds, browse, and tooth wear: a sheep perspective
Source: Ecol Evol. 2016 Jul 14;6(16):5559–69. doi: 10.1002/ece3.2241 (PMC4983574; doi:10.1002/ece3.2241)

Chestnut group
Diet: 75% clover + 25% chestnuts

Photo simulation - 07823-M2-U-L-TOT

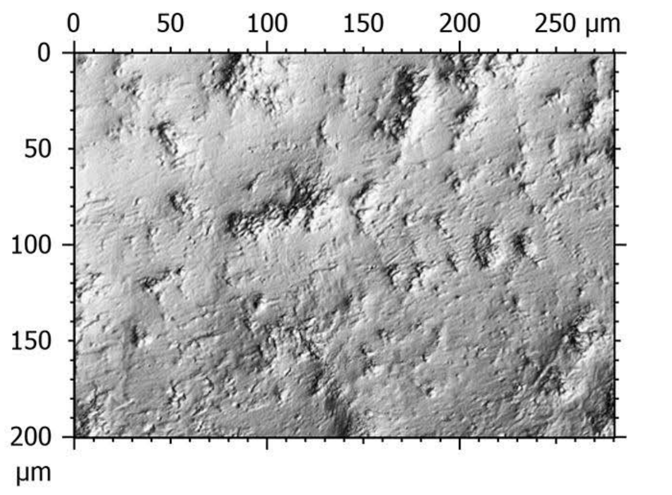

Photo simulation - 20251-M2-U-L-TOT

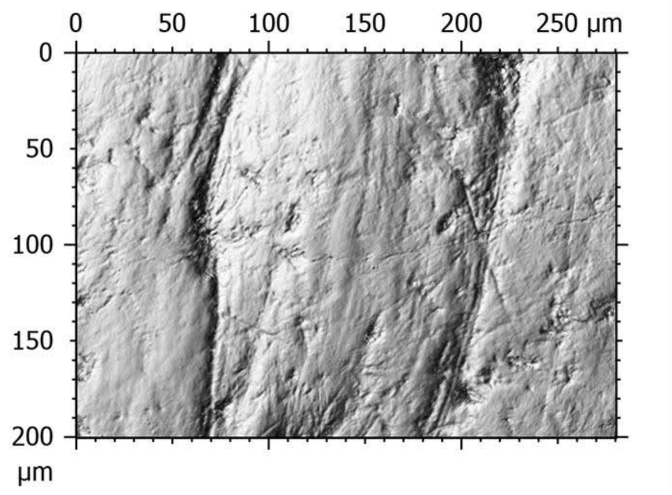

Photo simulation - 21269-M2-U-L-quad-TOT

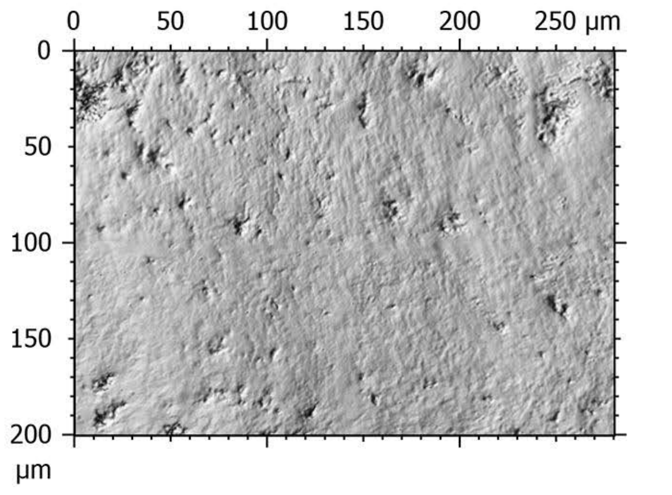

Photo simulation - 30003-M2-U-L-bis-TOT

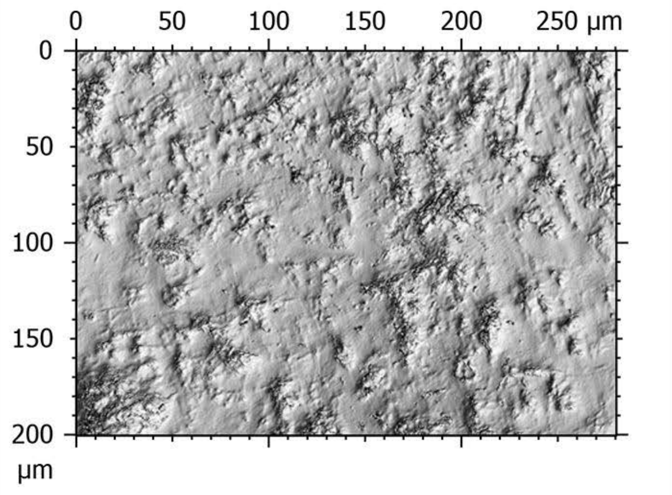

Photo simulation - 30870-M2-U-L-5-TOT

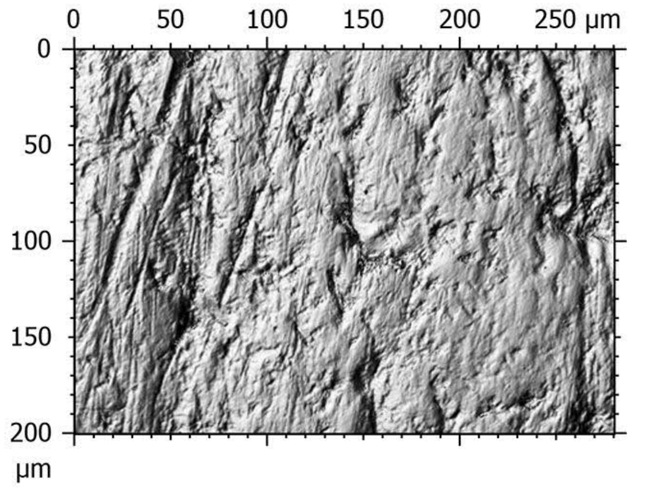

Photo simulation - 31051-M2-U-L-bis-TOT

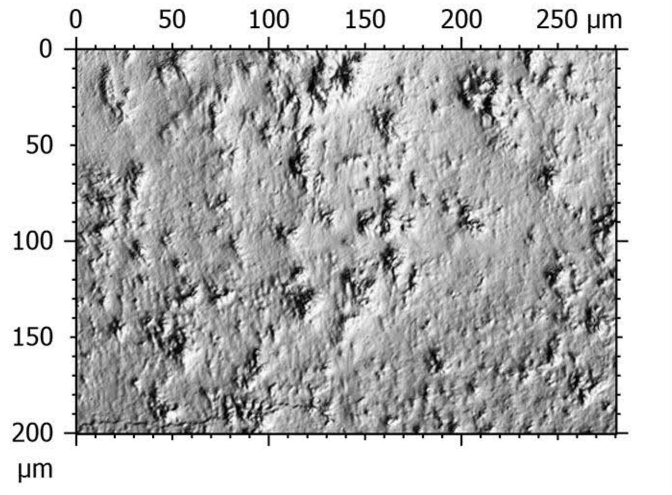

Leica Map DCM 3D 7.0.6863

Chestnut group
Diet: 75% clover + 25% chestnut

Photo simulation - 80171-M2-U-L-ter-TOT

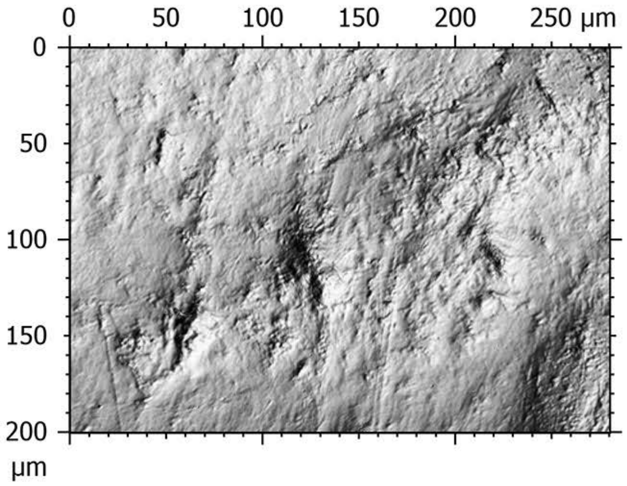

Photo simulation - 80212-M2-U-L-TOT

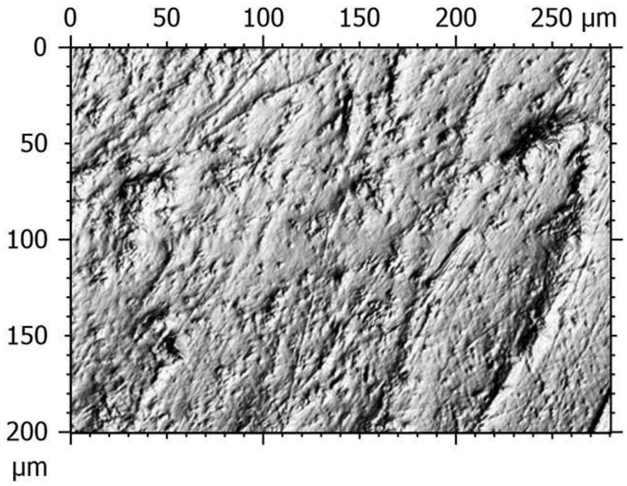

Photo simulation - 80721-M2-U-L-bis-TOT

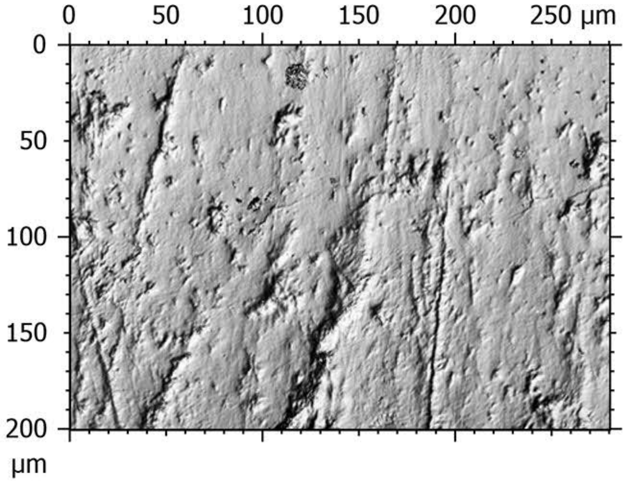

Photo simulation - 90126-M2-U-L-TOT

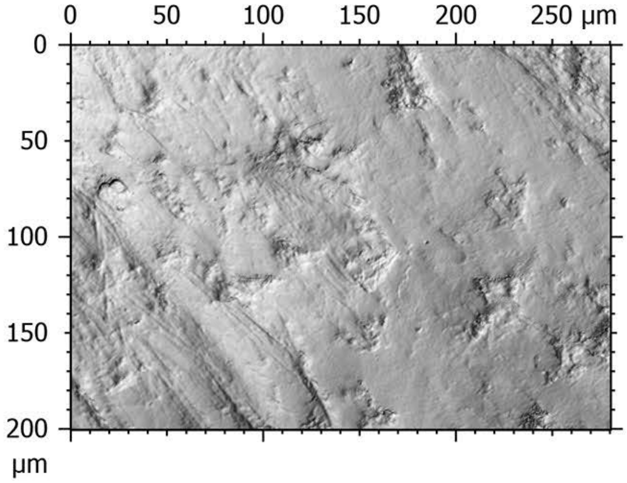

Leica Map DCM 3D 7.0.6863

Clover group
Diet: 100% clover

Photo simulation - L5-08-045-U-L-M2

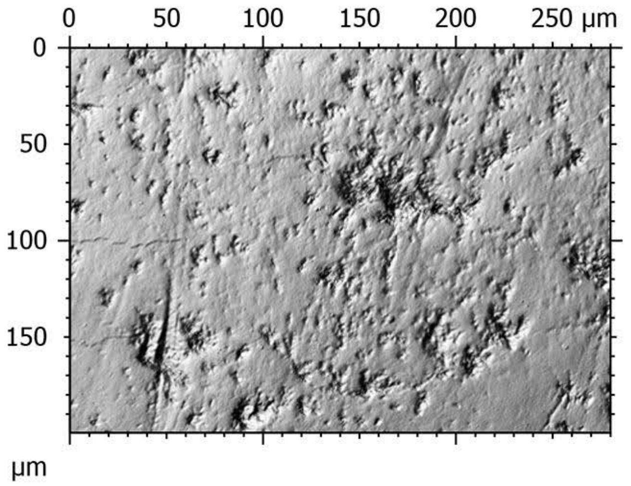

Photo simulation - L5-10-098-U-L-M2

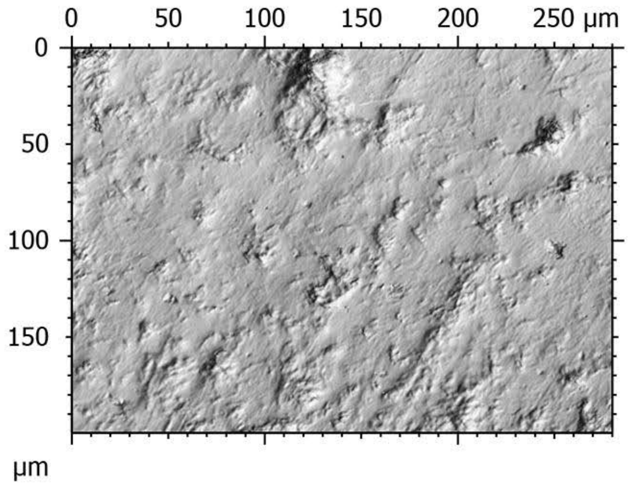

Photo simulation - L5-11-723-U-L-M2

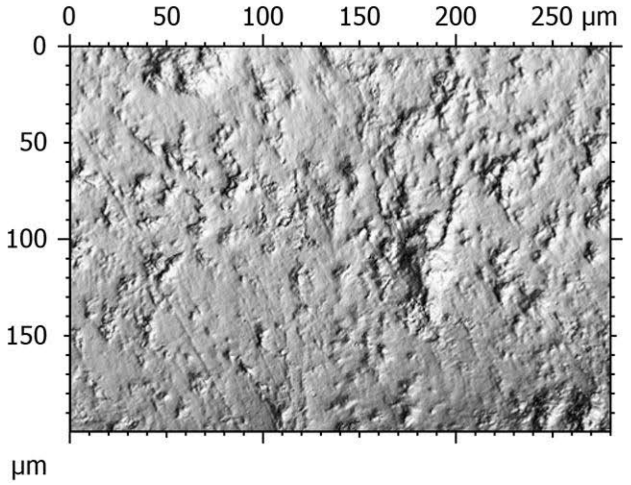

Photo simulation - L5-20-939-U-L-M2

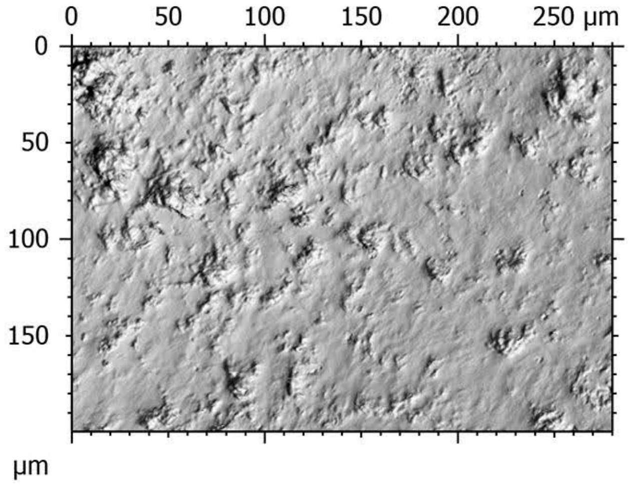

Photo simulation - L5-31-042-U-L-M2

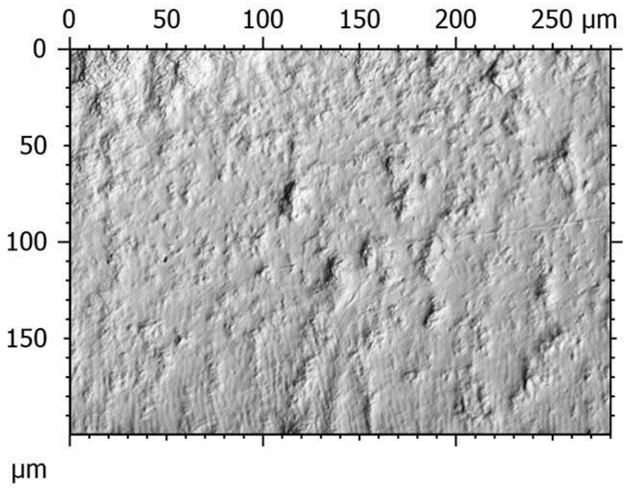

Photo simulation - L5-70-519-U-L-M2

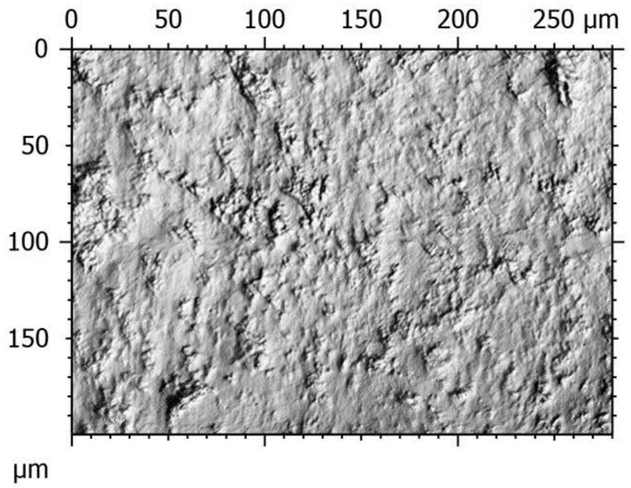

Leica Map DCM 3D 7.0.6863

Clover group
Diet: 100% clover

Photo simulation - L5-80-140-U-L-M2

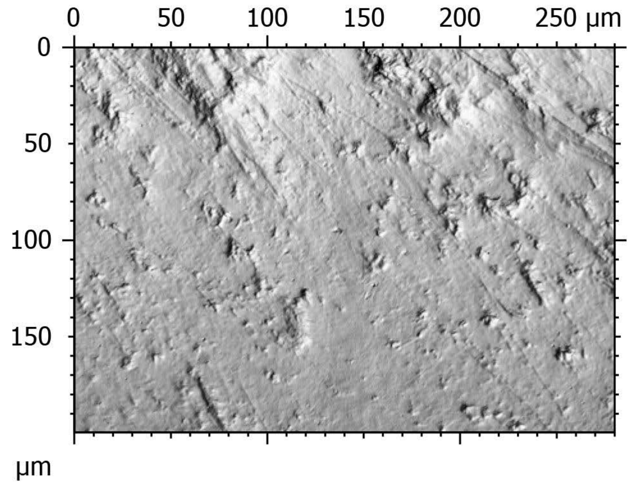

Photo simulation - L5-80-307-U-L-M2

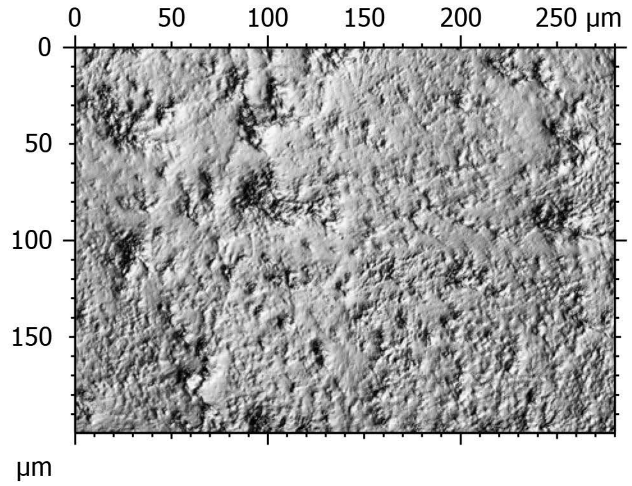

Photo simulation - L5-80-729-U-L-M2

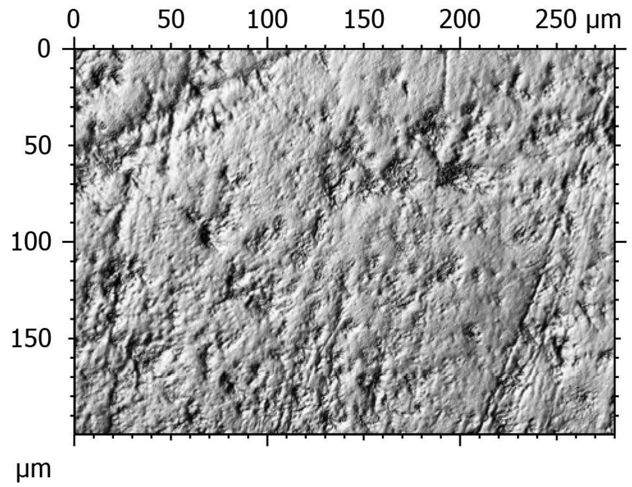

Photo simulation - L5-90-287-U-L-M2

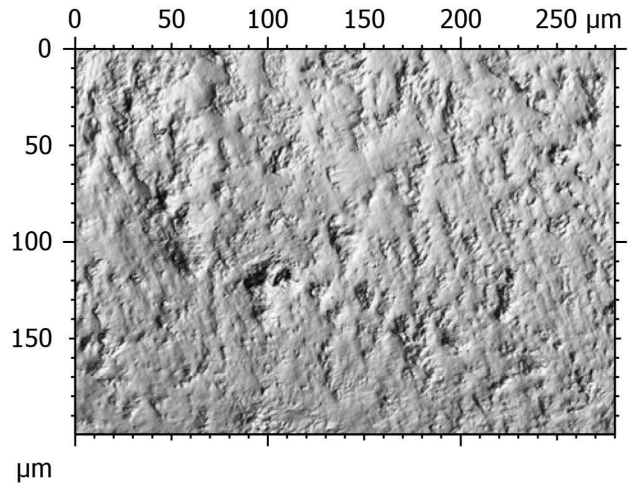

Leica Map DCM 3D 7.0.6863

Trident ANR

Barley group
Diet: 75% clover + 25% barley

Photo simulation - LO-Zinv-07347-U-L-M2-bis-TOT

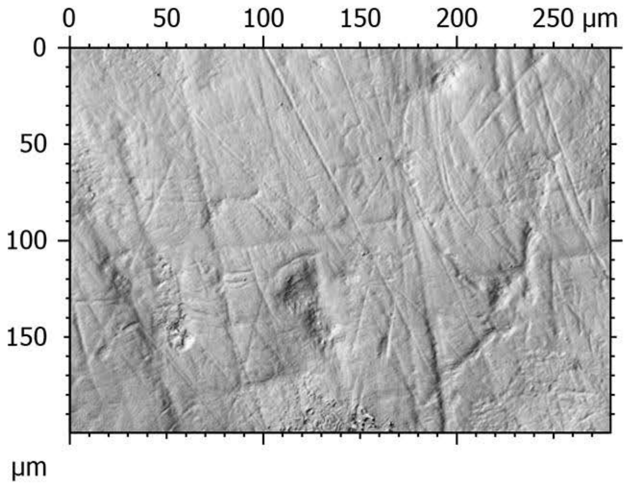

Photo simulation - LO-Zinv-30068-U-L-M2-TOT

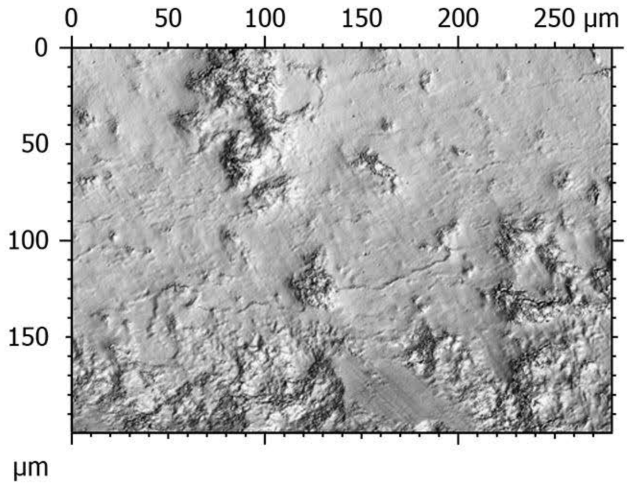

Photo simulation - LO-Zinv-21253-U-L-M2-TOT

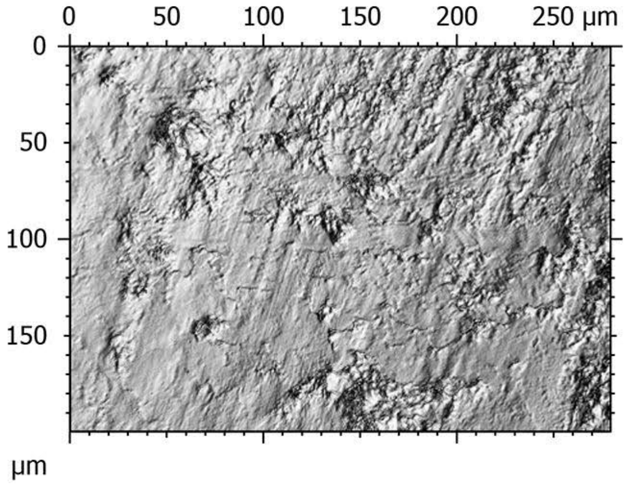

Photo simulation - LO-Zinv-31045-U-L-M2-TOT

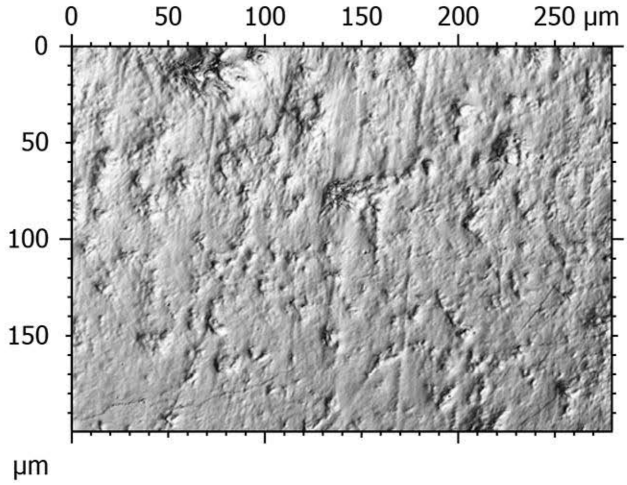

Photo simulation - LO-Zinv-21745-U-L-M2-ter-TOT

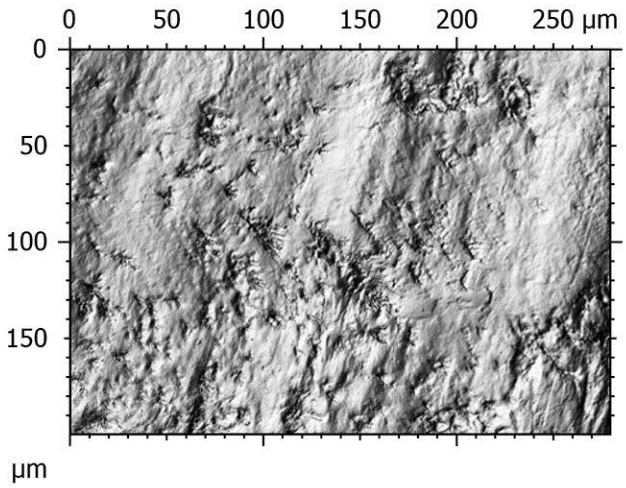

Photo simulation - LO-Zinv-31078-U-L-M2-TOT

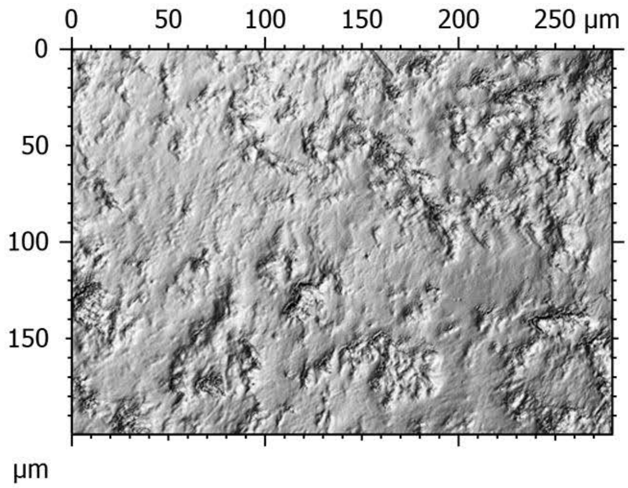

Leica Map DCM 3D 7.0.6863

Barley group
Diet: 75% clover + 25% barley

Photo simulation - LO-Zinv-80012-bis-U-L-M2-TOT

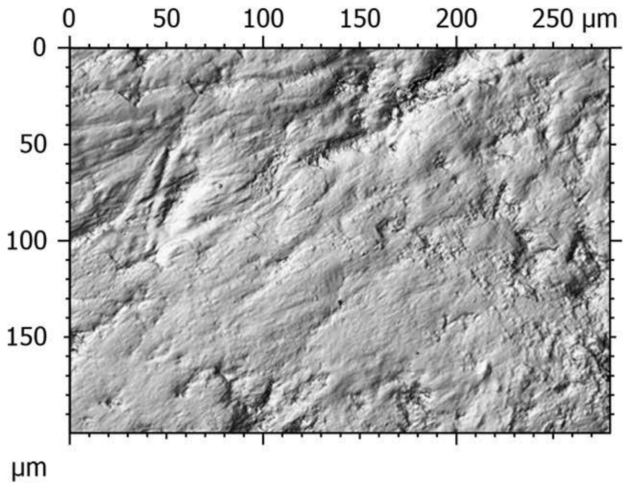

Photo simulation - LO-Zinv-80369-U-L-M2-bis-TOT

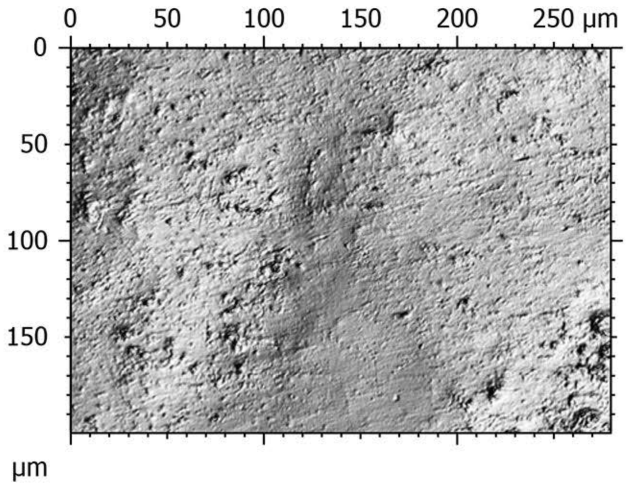

Photo simulation - LO-Zinv-80661-U-L-M2-bis-TOT

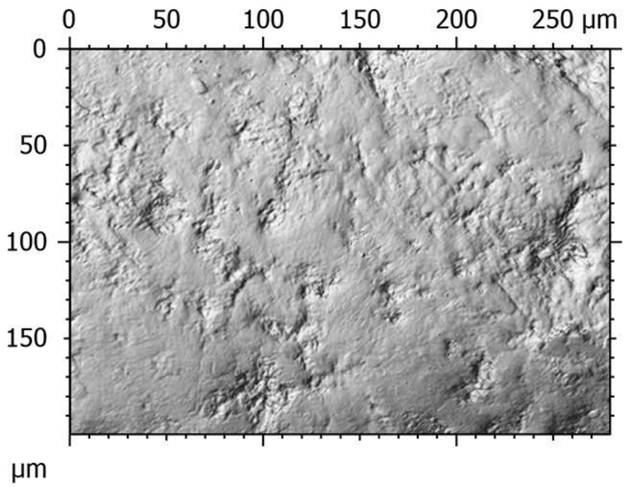

Photo simulation - LO-Zinv-90237-U-L-M2-ter-TOT

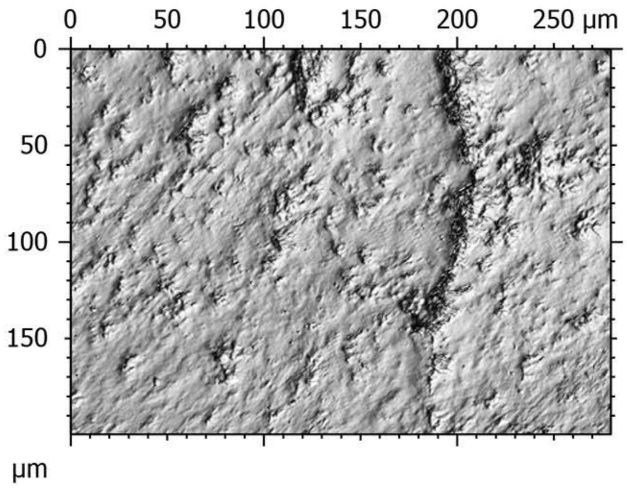

Corn group
Diet: 75% clover + 25% corn

Photo simulation - LM-Zinv-00063-L-M2-TOT

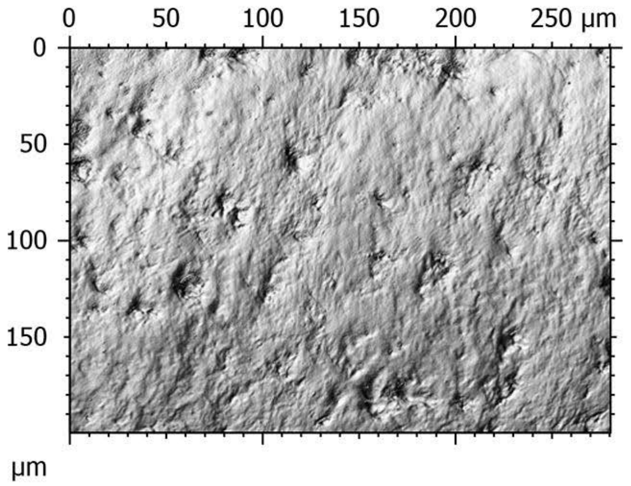

Photo simulation - LM-Zinv-21150-L-M2-TOT

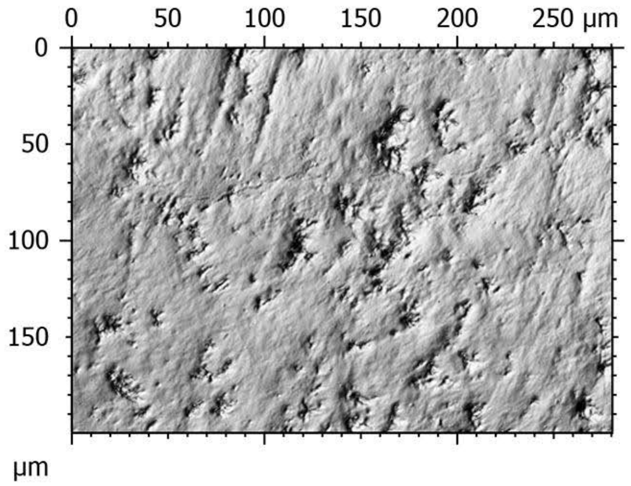

Photo simulation - LM-Zinv-21315-L-M2-ter-TOT

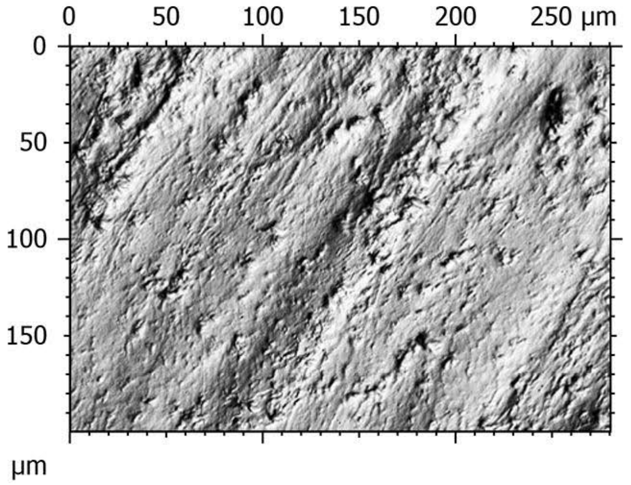

Photo simulation - LM-Zinv-30017-L-M2-bis-TOT

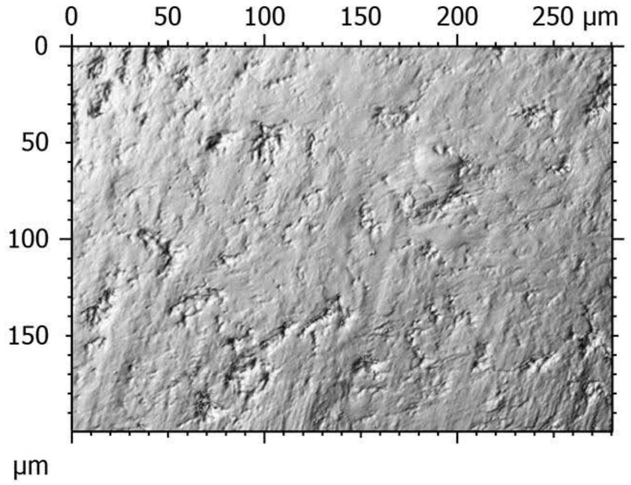

Photo simulation - LM-Zinv-31033-L-M2-ter-TOT

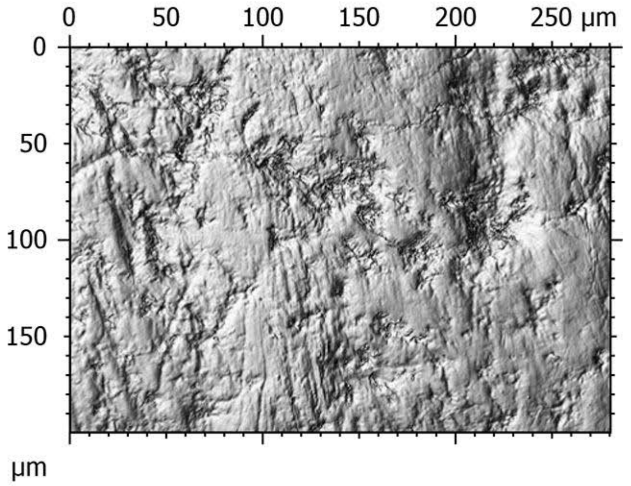

Photo simulation - LM-Zinv-31075-L-M2-TOT

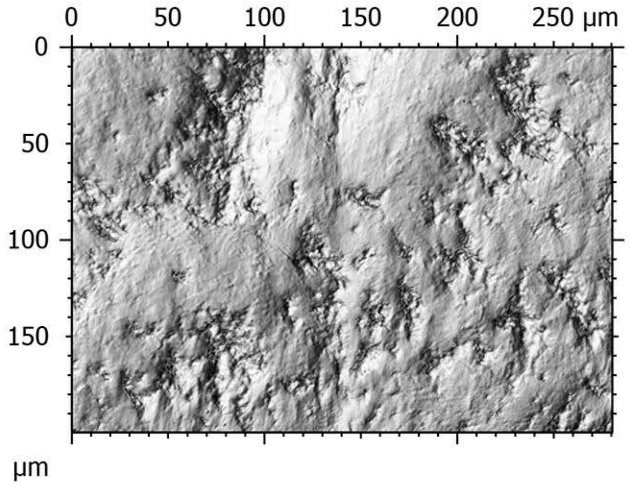

Leica Map DCM 3D 7.0.6863

Corn group
Diet: 75% clover + 25% corn

Photo simulation - LM-Zinv-80086-L-M1-TOT

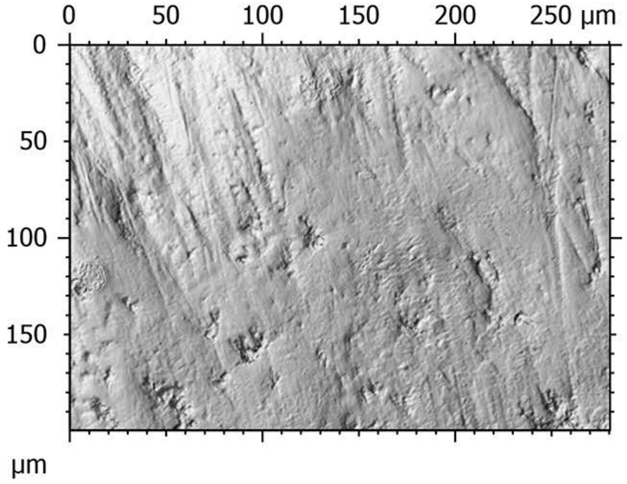

Photo simulation - LM-Zinv-80236-L-M2-ter-TOT

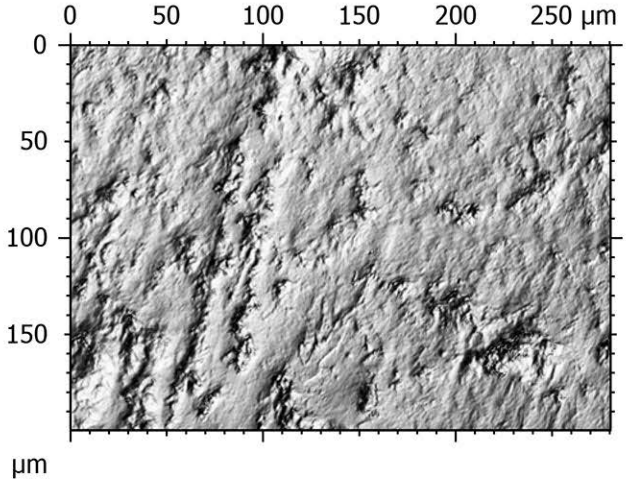

Photo simulation - LM-Zinv-80403-L-M2-bis-TOT

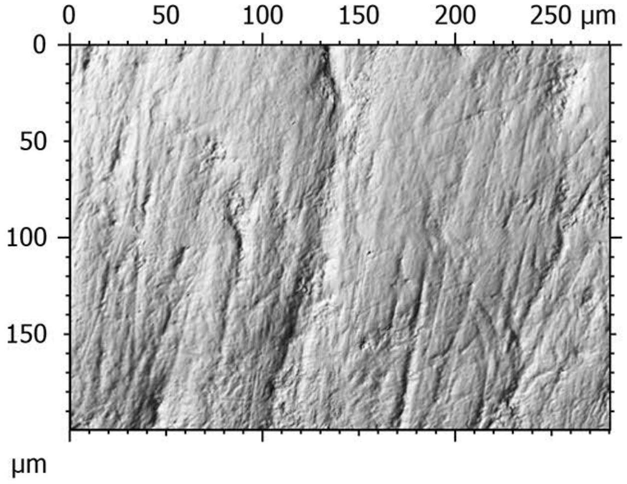

Photo simulation - LM-Zinv-90171-L-M2-ter-TOT

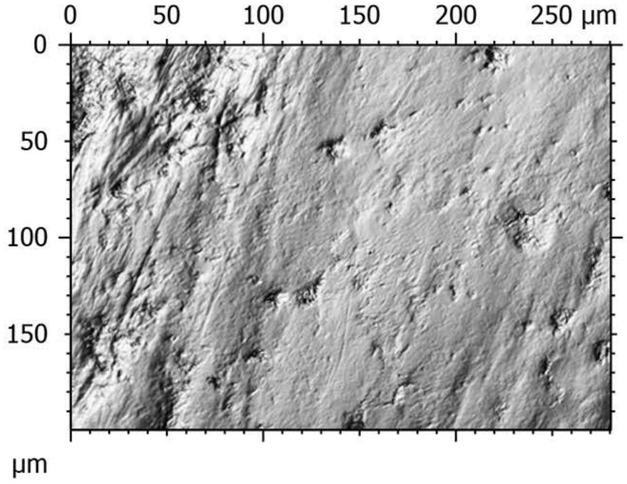

Supplement: Supplementary file 2 — Appendix S2. Photosimulations of the enamel surfaces scanned with the Leica DCM8 surface profilometer for each of the 40 ewes. [file ECE3-6-5559-s002.pdf]
