# Supplementary material for: Seeds, browse, and tooth wear: a sheep perspective
Source: Ecol Evol. 2016 Jul 14;6(16):5559–69. doi: 10.1002/ece3.2241 (PMC4983574; doi:10.1002/ece3.2241)
Supplement: Supplementary file 3 — Appendix S3. Asfc distribution according to specimen age and according to dietary group: clover (in green); chestnuts (in red); corn (in blue); barley (in black). [file ECE3-6-5559-s003.docx]

Appendix S3. Asfc distribution according to specimen age and to dietary group: clover (in green); chestnuts (in red); corn (in blue); barley (in black).

Asfc

Age
