# Supplementary material for: Seeds, browse, and tooth wear: a sheep perspective
Source: Ecol Evol. 2016 Jul 14;6(16):5559–69. doi: 10.1002/ece3.2241 (PMC4983574; doi:10.1002/ece3.2241)
Supplement: Supplementary file 4 — Appendix S4. Canonical discriminant analysis. Posterior individual probabilities for the CDA comparing clover‐fed and seed‐fed ewes (a) and comparing the four dietary categories (b). [file ECE3-6-5559-s004.docx]

Appendix S4. Canonical Discriminant Analysis. Posterior individual probabilities (without Jackknife resampling procedures) for the CDA comparing clover-fed and seed-fed ewes (a) and comparing the four dietary categories (b). Highest probabilities are in bold.

(a)

|  |  |  | Posterior individuals probability to be classified into the | |
| --- | --- | --- | --- | --- |
| Class | Group | Specimen | Clover class | Seeds class |
| Clover | Clover | 08-045 | **0.673** | 0.327 |
| Clover | Clover | 10-098 | **0.628** | 0.372 |
| Clover | Clover | 11-723 | **0.732** | 0.268 |
| Clover | Clover | 20-939 | **0.715** | 0.285 |
| Clover | Clover | 31-042 | **0.633** | 0.367 |
| Clover | Clover | 70-519 | 0.392 | **0.608** |
| Clover | Clover | 80-140 | 0.358 | **0.642** |
| Clover | Clover | 80-307 | 0.134 | **0.866** |
| Clover | Clover | 80-729 | 0.449 | **0.551** |
| Clover | Clover | 90-287 | **0.593** | 0.407 |
| Seeds | Chestnuts | 07-823 | 0.070 | **0.930** |
| Seeds | Chestnuts | 20-251 | 0.018 | **0.982** |
| Seeds | Chestnuts | 21-269 | 0.075 | **0.925** |
| Seeds | Chestnuts | 30-003 | 0.023 | **0.977** |
| Seeds | Chestnuts | 30-870 | 0.052 | **0.948** |
| Seeds | Chestnuts | 31-051 | **0.558** | 0.442 |
| Seeds | Chestnuts | 80-171 | 0.154 | **0.846** |
| Seeds | Chestnuts | 80-212 | 0.204 | **0.796** |
| Seeds | Chestnuts | 80-721 | 0.092 | **0.908** |
| Seeds | Chestnuts | 90-126 | 0.015 | **0.985** |
| Seeds | Corn | 00-063 | 0.034 | **0.966** |
| Seeds | Corn | 21-150 | 0.207 | **0.793** |
| Seeds | Corn | 21-315 | **0.532** | 0.468 |
| Seeds | Corn | 30-017 | 0.144 | **0.856** |
| Seeds | Corn | 31-033 | 0.068 | **0.932** |
| Seeds | Corn | 31-075 | 0.032 | **0.968** |
| Seeds | Corn | 80-086 | 0.237 | **0.763** |
| Seeds | Corn | 80-236 | 0.447 | **0.553** |
| Seeds | Corn | 80-403 | 0.093 | **0.907** |
| Seeds | Corn | 90-171 | 0.060 | **0.940** |
| Seeds | Barley | 07-347 | 0.041 | **0.959** |
| Seeds | Barley | 21-253 | 0.068 | **0.932** |
| Seeds | Barley | 21-745 | 0.080 | **0.920** |
| Seeds | Barley | 30-068 | 0.000 | **1.000** |
| Seeds | Barley | 31-045 | **0.561** | 0.439 |
| Seeds | Barley | 31-078 | 0.056 | **0.944** |
| Seeds | Barley | 80-012 | 0.089 | **0.911** |
| Seeds | Barley | 80-369 | 0.297 | **0.703** |
| Seeds | Barley | 80-661 | 0.156 | **0.844** |
| Seeds | Barley | 90-237 | 0.313 | **0.687** |

(b)

|  |  | Posterior individuals probabilities to be classified into the | | | |
| --- | --- | --- | --- | --- | --- |
| Class/Group | Specimen | Clover class | Chestnut class | Corn class | Barley class |
| Clover | 08-045 | **0.708** | 0.096 | 0.172 | 0.025 |
| Clover | 10-098 | **0.702** | 0.082 | 0.142 | 0.075 |
| Clover | 11-723 | **0.742** | 0.019 | 0.097 | 0.142 |
| Clover | 20-939 | **0.732** | 0.036 | 0.069 | 0.163 |
| Clover | 31-042 | **0.689** | 0.083 | 0.187 | 0.041 |
| Clover | 70-519 | 0.367 | **0.379** | 0.236 | 0.018 |
| Clover | 80-140 | **0.393** | 0.198 | 0.373 | 0.036 |
| Clover | 80-307 | 0.177 | 0.109 | **0.563** | 0.151 |
| Clover | 80-729 | **0.472** | 0.209 | 0.292 | 0.027 |
| Clover | 90-287 | **0.648** | 0.120 | 0.194 | 0.038 |
| Chestnuts | 07-823 | 0.090 | 0.218 | **0.627** | 0.065 |
| Chestnuts | 20-251 | 0.015 | **0.693** | 0.280 | 0.012 |
| Chestnuts | 21-269 | 0.082 | **0.535** | 0.345 | 0.038 |
| Chestnuts | 30-003 | 0.005 | 0.015 | 0.009 | **0.972** |
| Chestnuts | 30-870 | 0.076 | **0.475** | 0.300 | 0.150 |
| Chestnuts | 31-051 | **0.601** | 0.193 | 0.170 | 0.036 |
| Chestnuts | 80-171 | 0.182 | 0.196 | **0.569** | 0.053 |
| Chestnuts | 80-212 | 0.148 | **0.673** | 0.162 | 0.017 |
| Chestnuts | 80-721 | 0.061 | **0.777** | 0.144 | 0.018 |
| Chestnuts | 90-126 | 0.013 | **0.693** | 0.276 | 0.018 |
| Corn | 00-063 | 0.033 | **0.571** | 0.375 | 0.021 |
| Corn | 21-150 | 0.204 | **0.470** | 0.298 | 0.027 |
| Corn | 21-315 | **0.460** | 0.341 | 0.190 | 0.009 |
| Corn | 30-017 | 0.168 | 0.087 | **0.653** | 0.093 |
| Corn | 31-033 | 0.080 | 0.074 | 0.256 | **0.590** |
| Corn | 31-075 | 0.049 | 0.335 | 0.228 | **0.388** |
| Corn | 80-086 | 0.263 | 0.216 | **0.484** | 0.037 |
| Corn | 80-236 | **0.524** | 0.067 | 0.246 | 0.164 |
| Corn | 80-403 | 0.082 | 0.443 | **0.462** | 0.013 |
| Corn | 90-171 | 0.069 | 0.280 | **0.616** | 0.035 |
| Barley | 07-347 | 0.060 | 0.332 | **0.520** | 0.089 |
| Barley | 21-253 | 0.077 | 0.119 | 0.159 | **0.645** |
| Barley | 21-745 | 0.049 | 0.091 | 0.041 | **0.820** |
| Barley | 30-068 | 0.000 | 0.090 | 0.009 | **0.901** |
| Barley | 31-045 | **0.571** | 0.041 | 0.109 | 0.278 |
| Barley | 31-078 | 0.027 | 0.049 | 0.033 | **0.891** |
| Barley | 80-012 | 0.056 | 0.064 | 0.054 | **0.826** |
| Barley | 80-369 | 0.335 | 0.087 | **0.505** | 0.073 |
| Barley | 80-661 | 0.160 | 0.051 | 0.212 | **0.577** |
| Barley | 90-237 | 0.057 | 0.010 | 0.006 | **0.927** |
